# Sorting Mechanisms for MicroRNAs into Extracellular Vesicles and Their Associated Diseases

**DOI:** 10.3390/cells9041044

**Published:** 2020-04-22

**Authors:** Michael Groot, Heedoo Lee

**Affiliations:** 1 Department of Medicine, Boston University Medical Campus, Boston, MA 02118, USA; mgroot@bu.edu; 2 Department of Biology and Chemistry, Changwon National University, Changwon 51140, Korea

**Keywords:** extracellular vesicle, exosome, microvesicle, microRNA, RNA-binding protein

## Abstract

Extracellular vesicles (EV) are secretory membranous elements used by cells to transport proteins, lipids, mRNAs, and microRNAs (miRNAs). While their existence has been known for many years, only recently has research begun to identify their function in intercellular communication and gene regulation. Importantly, cells have the ability to selectively sort miRNA into EVs for secretion to nearby or distant targets. These mechanisms broadly include RNA-binding proteins such as hnRNPA2B1 and Argonaute-2, but also membranous proteins involved in EV biogenesis such as Caveolin-1 and Neural Sphingomyelinase 2. Moreover, certain disease states have also identified dysregulated EV-miRNA content, shedding light on the potential role of selective sorting in pathogenesis. These pathologies include chronic lung disease, immune response, neuroinflammation, diabetes mellitus, cancer, and heart disease. In this review, we will overview the mechanisms whereby cells selectively sort miRNA into EVs and also outline disease states where EV-miRNAs become dysregulated.

## 1. Introduction

EVs are a broad group of membranous vesicles classified based on size, function, RNA profiles, or method of biogenesis. Based on classifications from the International Society of Extracellular Vesicles, EVs can be subdivided into exosomes, microvesicles (MVs), and apoptotic bodies (ABs) [1]. Regardless of the subtype, EVs are gaining increased attention for their function in transporting both proteins and RNA extracellularly for wide-ranging effects [2,3]. In particular, this exosome-mediated transfer of mRNA and microRNA (miRNA) has been shown to induce effects on recipient cells, such as regulate protein expression, suggesting an in vivo functional role of exosome-derived mRNA and miRNA [4]. To achieve these regulatory functions, the RNA and miRNA content of EVs is markedly different from the RNA content of the parent cell, meaning that cells are capable of increasing or decreasing the concentration of EV RNAs [5,6,7]. Furthermore, certain populations of miRNA-rich EVs have been identified that represent 6% of the total EVs but approximately 39% of the total EV-derived RNA [8]. Collectively, this evidence supports the concept that miRNAs can be selectively sorted into EVs through a purposeful rather than passive process. Thus far, research has identified several miRNA sorting mechanisms, but the specific details remain incompletely understood.

In this review, we will attempt to overview the current understanding of the miRNA sorting processes into EVs while demonstrating the areas still in need of further research. Specifically, we will identify the miRNAs involved in each sorting process and the underlying proteins driving each mechanism. This will be divided into a description of RNA-binding protein (RBP) mechanisms as well as membranous proteins involved in EV biogenesis. Finally, we will discuss the effect of certain disease states such as heart disease and diabetes mellitus (DM) on miRNA packaging.

## 2. RNA-Binding Proteins

### 2.1. Heterogeneous Nuclear Ribonucleoproteins

**Figure 1:**
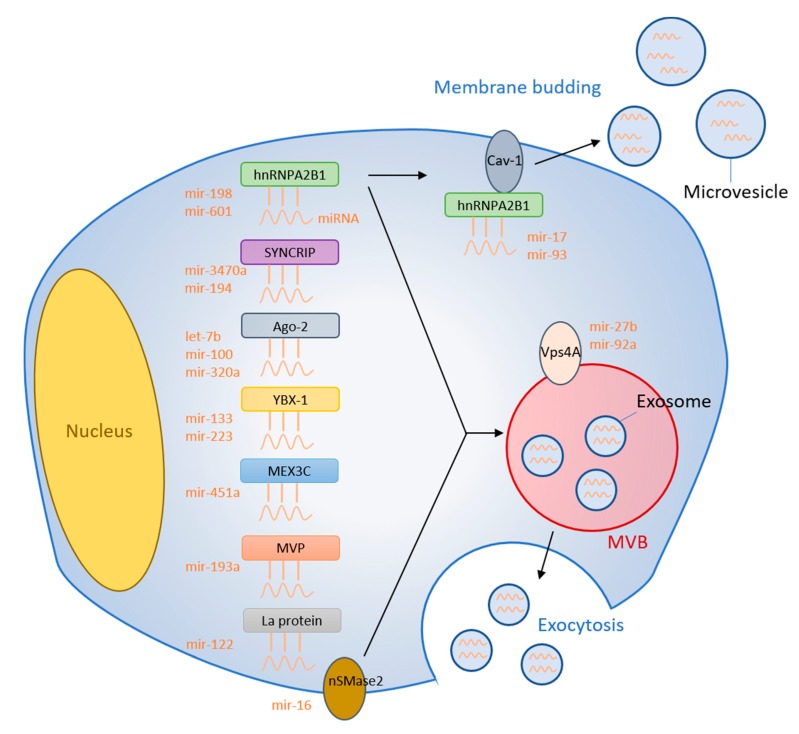
Summary of miRNA sorting into EVs. RNA binding proteins bind specific miRNAs and selectively shuttle them into EVs. Membranous proteins are also involved in the miRNA sorting mechanism.

RBPs bind specific RNA molecules to assist with the sorting process into exosomes. Heterogeneous nuclear ribonucleoprotein A2B1 (hnRNPA2B1) is one such RNA binding protein capable of targeting miRNAs to control the loading into exosomes [9]. The hnRNPA2B1 protein was found to undergo sumoylation and bind miRNA-198 [9]. This binding interaction then localizes the hnRNPA2B1-miRNA-198 complex into exosomes for extracellular transport (Figure 1). Interestingly, Villarroya-Beltri et al. identified GGAG/UGCA motifs for the interaction between hnRNPA2B1 and miR-198 and -601 [9]. Another report showed that AGG/UAG motifs are specifically recognized by hnRNPA2B1 and Lee et al. demonstrated that hnRNPA2B1 strongly interacts with MV-associated miRNA-17 and -93, which both have AGG/UAG motifs [10,11]. These binding motifs could provide a potential mechanism whereby hnRNPA2B1 exerts regulatory control over miRNA sorting. Another RBP, heterogeneous nuclear ribonucleoprotein A1 (hnRNPA1), also appears to demonstrate binding affinity to miRNA-198. It becomes sumoylated in a similar fashion to hnRNPA2B1, suggesting a potential role of sorting miRNA into EVs [12]. While these collective findings have demonstrated a positive interaction between hnRNPs and miRNA loading into exosomes, other reports have specifically shown a negative interaction between hnRNPA2B1 and miR-503 [13]. Knockdown of hnRNPA2B1 correlated with increased miR-503 levels within exosomes. Importantly, miR-503 does not contain any motifs known to bind hnRNPA2B1. This suggests that hnRNPA2B1 could be involved in shuttling certain miRNAs into exosomes via specific RNA motifs while selectively preventing the addition of other miRNAs via another unidentified mechanism.

Synaptotagmin-binding cytoplasmic RNA-interaction protein (SYNCRIP) is another member of the hnRNP protein family and is also known as hnRNP-Q. SYNCRIP was found to associate and precipitate with miR-3470a and miR-194-2-3p, which are both miRNAs highly enriched in exosomes [14]. SYNCRIP was not found to associate with cytoplasmic miRNAs or other random sequences, suggesting a high level of specificity for the exosome-derived miRNAs. Further, shRNAs against SYNCRIP caused a reduction in the miRNA levels within exosomes and an increase in the cytoplasmic miRNA [14]. These findings suggest that SYNCRIP is involved in loading specific miRNAs into exosomes, and deficiency in SYNCRIP causes accumulation of miRNA in the cytosol. As a potential mechanism for this selective miRNA sorting, SYNCRIP was capable of binding a GGCU sequence found in certain exosome-enriched miRNAs, such as miR-3470a and miR-194-2-3p [14]. Further work has identified that SYNCRIP contains an N-terminal unit for RNA recognition (NURR) domain capable of binding to the GGCU sequence contained within miR-3470 and other exosome-associated miRNAs [15]. Removal of the NURR domain from SYNCRIP impairs the SYNCRIP-miR-3470 binding, providing further evidence that these domains are critical in exosome loading.

### 2.2. Argonaute 2

Cells exert control over endogenous mRNA through RNA interference (RNAi) using proteins such as Drosha, Dicer, and RISC. The argonaute2 (Ago2) protein is a component of the RISC complex that binds miRNAs and facilitates mRNA degradation through endonuclease activity. Recent findings have shown the presence of circulating Ago2-miRNA complexes in human plasma, which suggests that Ago2 might have an important role in the stability of secreted miRNA [16]. Additionally, Ago2 has been identified within exosomes and has separately been shown to protect miRNA contained within MVs from RNase degradation [17]. Expectedly, Ago2 has also been implicated in binding and sorting miRNA into EVs through the KRAS-MEK-ERK signaling pathway [18]. Specifically, phosphorylated Ago2 inhibits its co-localization with MVs and decreases secretion into endosomes. Conversely, inhibition of MEK and ERK was shown to decrease Ago2 phosphorylation and increase Ago2 accumulation inside exosomes [18]. Thus, the miRNA sorting exerted by Ago2 appears to be controlled upstream by the KRAS-MEK-ERK pathway. Further, KRAS-MEK-ERK pathway-dependent phosphorylation of Ago2 has been demonstrated to exert some specific control over the sorting of let-7a, miR-100, and miR-320a into exosomes [18]. Strengthening these findings, miR-100 levels within exosomes are elevated in oncogenic KRAS mutants with overactive phosphorylation [19]. Collectively, KRAS-MEK-ERK has general regulatory ability over MV and exosomal levels of Ago2, in addition to specific control over select miRNAs.

### 2.3. Y-Box Binding Protein 1

Y-Box Binding Protein 1 (YBX-1) is another protein with RNA-binding domains and exerts a range of functions including mRNA splicing and transport [20,21]. More recently, YBX-1 has been found to regulate miR-133 packaging into exosomes after hyperoxia/reperfusion (H/R) treatment of endothelial progenitor cells (EPC) [22]. Silencing YBX-1 through siRNA causes decreased miR-133 localization within H/R EPC-derived exosomes with no change in expression in the EPC cytosol. Conversely, overexpression of YBX-1 with miR-133 mimics increased miR-133 quantity within H/R EPC-derived exosomes.

Additionally, YBX-1 was also identified through liquid chromatography and mass spectrometry as a candidate for binding miR-223 [23]. From this identification, it was found that YBX-1 was required to selectively package miR-223 into exosomes derived from HEK293T cells. To confirm that the effect of YBX-1 is selective to miR-223, they also evaluated the influence of YBX-1 over miR-190 packaging. miR-190 is not normally known to be present in exosomes, and YBX-1 indeed produced no change in miR-190 presence within exosomes, suggesting that YBX-1 does have a selective effect on miR-223. As mentioned, YBX-1 contains RNA-binding domains to achieve its endogenous functions within the cell, so it likely interacts with miR-133 and miR-223 through specific RNA-binding domains.

### 2.4. MEX3C

MEX3C functions as an RNA-binding E3 ubiquitin ligase to assist with mRNA degradation. However, recent evidence has suggested that MEX3C could also play a role in miRNA sorting into EVs [24]. Immunoprecipitation experiments demonstrate that MEX3C associates with Ago2, which has been shown in this review to be involved in miRNA sorting [24]. Recently, MEX3C has also been shown to co-localize with adaptor-related protein complex 2 (AP-2), which is an adaptor protein implicated in clathrin-mediated endocytosis [25]. As endosomes are processed by the cell, they form into multivesicular bodies (MVB). Exosomes are then formed when MVBs fuse with the plasma membranes and release their cargo proteins and RNAs. Given the MEX3C-endosome and MEX3C-Ago2 relationships, it is reasonable to predict that MEX3C exerts some influence over exosome biogenesis and potential miRNA sorting. In an additional experiment, siRNA molecules targeting MEX3C cause decreased exosomal miR-451a [25]. This study found no complementary sequences between MEX3C and miR-451a, so the authors suggested that Ago2 might serve as the intermediary between MEX3C and miR-451a. Collectively, this MEX3C-Ago2 complex could shuttle miR-451a into exosomes.

### 2.5. Major Vault Protein

Major vault protein (MVP) is a ribonucleoprotein involved in transporting RNA from the nucleus to the cytoplasm [26]. Recently, MVP has also been proposed as a regulator of miRNAs sorting into exosomes [27]. MVP knockout in CT26 colon cancer cells caused increased cellular miR-193a, but decreased levels within CT26-derived exosomes [27]. This MVP knockout was miR-193a selective because there were no observed expression changes in another tested miRNA, miR-126a. Expectedly, MVP overexpression in CT26 cells caused reduced miR-193a levels within the cytosol and increased exosomal concentration [27]. To further strengthen these findings, MVP was identified as a potential binding partner of miR-193a through mass spectrometry [27]. Collectively, these findings indicate that miR-193a is sorted into exosomes via an MVP-dependent process, but the specific mechanism is still unknown.

### 2.6. La Protein

The La protein is an RNA-binding protein that functions as a transcription factor for RNA polymerase III that shuttles between the nucleus and cytoplasm [28]. La-depleted cytosol was associated with a 4-fold reduction of miR-122 sequestration into EVs [29]. However, miR-122 levels returned to normal with the addition of La to the cytosol [29]. Importantly, the addition of La exclusively without cytosol produced no effects, indicating the necessity of other unknown cytoplasmic proteins in the mechanism. Furthermore, electrophoretic mobility shift assays demonstrated a higher binding affinity between La and miR-122 (K_d_ = 4.8 nM) than another RNA-binding protein, Ago2 (K_d_ = 10 nM) [29]. An overview of this mechanism and all other RNA-binding proteins is presented in Figure 1 and Table 1.

## 3. Membrane Proteins

### 3.1. Caveolin-1

Caveolae are small 50 to 100nm invaginations of the plasma membrane that assist with receptor-independent endocytosis and exocytosis [30]. Caveolin-1 (Cav-1) proteins are localized within these caveolae and demonstrate a critical role in the regulation of membrane trafficking. Under periods of cell stress, such as during hyperoxia and ROS generation, Cav-1 becomes upregulated in MV membranes [11]. Furthermore, Cav-1 overexpression caused increased release of hnRNPA2B1 into MVs as well as elevated levels of hnRNPA2B1-associated miRNAs in MVs of hyperoxia-treated cells [11]. Cav-1 deletion caused decreased expression of hnRNPA2B1 in MVs in response to hyperoxia [11]. Collectively, these results suggest that Cav-1 is integral in the trafficking process of hnRNPA2B1 and hnRNPA2B1-associated miRNAs into MVs. Additionally, Cav-1 has been identified in populations of miRNA-rich EVs derived from lung epithelial cells [8]. This further strengthens the concept that Cav-1 plays an important role in selective transport of miRNAs into EVs.

Low molecular weight hyaluronan (LMW-HA) is another plasma membrane component and is known to cause ROS production inside cells. Treating cells with LMW-HA causes time-dependent induction of exosome production [31]. However, this effect disappears when caveolin-enriched microdomains (CEM) are inhibited from forming. CEM are plasma membrane regions enriched with Cav-1 [32]. Thus, without the presence of Cav-1, exosome biogenesis appears to be impaired. These findings indicate that Cav-1 is a necessary component of EV biogenesis during ROS-induced cell stress.

### 3.2. Neural Sphingomyelinase 2

Neural Sphingomyelinase 2 (nSMase2) is a hydrolase involved in the metabolism of sphingolipids, which are a critical component of the plasma membrane. Specifically, it functions as the rate-limiting enzyme in ceramide biosynthesis [33]. Ceramide has been implicated in exosome biogenesis, making the nSMase2 enzyme a likely regulator of exosome synthesis by extension [34]. Inhibition of nSMase2 greatly reduced secretion of miR-16 and miR-146a within exosomes without any change in cellular miRNA levels [33]. Likewise, overexpression of nSMase2 increased expression of miR-16 and miR-146a in exosomes with no effect on cellular miRNA levels [33].

Human tumors have been shown to employ exosomes as a mode of signaling to nearby cells [35]. The nSMase2-dependent method of miRNA sorting also has potential implications in tumor signaling and progression. Exosome levels of miR-16 decreased significantly in nSMase2-knockdown mouse cancer cells but increased in nSMase2-overexpressing cancer cells [36]. nSMase2-knockdown cancer cells also demonstrated decreased lung metastatic colonization, with the nSMase2-overexpressing cells predictably increasing metastatic progression. In a separate study, inhibition of miR-16 has been shown to stimulate cell proliferation, suggesting that miR-16 has an important role in preventing proliferation and tumorigenesis [37]. Furthermore, apoptosis increased in tumor cells when transfected with pre-miR-16 when compared to controls [37]. Thus, the nSMase2-induced shuttling of miR-16 into exosomes could provide evidence for the theory that exosomes can be used as disposal mechanisms for unnecessary or unwanted mRNA or miRNA. Tumor cells could shuttle tumor suppressors such as miR-16 into exosomes for disposal via a nSMase2-dependent mechanism to prevent apoptosis and promote proliferation.

### 3.3. Vacuolar Protein Sorting-Associated Protein 4

The vacuolar protein sorting-associated protein 4 (Vps4A) is required for normal endosomal trafficking and MVB sorting, but has also recently been implicated in cancer [38,39]. Hepatocellular carcinoma cells (HCC) overexpressing Vps4A caused elevated exosomal levels of miR-27b-3p and miR-92a-3p, which are both known oncomiRs [40]. On the other hand, inhibition of Vps4A in HEK293 cells caused reduced levels of EV-derived miR-92a and miR-150 [41]. Interestingly, miR-27b-3p promotes migration and invasion of colorectal cancer cells while miR-92a-3p promotes proliferation, migration, and invasion of esophageal squamous cell cancer [42,43]. HCC cells overexpressing Vps4A also resulted in elevated levels of miR-193a-3p, miR-320a, and miR-132-3p, which are all known tumor suppressor miRNAs [40]. As evident by these results, Vps4A produces a tumor suppressor effect within the same cell but appears to secrete oncomiRs via exosomes that could potentially promote tumor migration in recipient other cells. More research needs to be conducted to determine the exact mechanism that Vps4A uses to sort miRNAs into EVs as well as determine its regulation over tumor growth. The membranous proteins that are involved in the miRNA sorting are graphically summarized in Figure 1.

## 4. Disease States

### 4.1. Chronic Lung Disease

The miRNAs contained within EVs originally come from the cytosol of the parent cell, meaning that there is a relationship between cytosolic and EV levels of miRNAs. As such, any condition or disease state that manipulates miRNA expression within individual cells can modulate the expression within EVs [44]. Thus, the miRNA content of EVs is thought to reflect the cellular response to stressors. Endogenously, certain cell stressors such as hyperoxia or hypoxia are widely known to vary the gene expression within a particular cell. Hyperoxia-treated lung epithelial cells caused an upregulation of miR-320a and miR-221 in MVs [45]. In another recent in vivo experiment by Go and colleagues, 2020, hyperoxia-treated mice developed increased levels of miR-21 in serum EVs [46]. These authors furthered their findings by measuring EV miRNA in serum derived from premature human infants as a model of chronic lung disease (CLD). Predictably based on the mouse experiments, they identified elevated serum EV levels of miR-21 in human infants [46]. These findings suggest that miR-21 is likely involved in the lung injury process associated with CLD. MicroRNA-21 has been previously shown to target and suppress the tumor suppressor protein Programmed Cell Death 4 (PDCD4) [47]. Go and colleagues, 2020, also identified that miR-21 suppressed PDCD4, and thus, suggested that the elevated serum EV miR-21 could be a potential biomarker of CLD [46].

### 4.2. Immune Response

Interleukin-4 (IL-4) is a cytokine known to activate IgE production and to aid the proliferation of helper T cells into Th-2 cells. Additionally, IL-4 has been shown to act on bone marrow-derived macrophages (BMDM) to produce differential expression of 40 miRNAs from BMDM-derived exosomes [48]. Of the differentially expressed miRNAs, miR-138-5p and miR-149-5p were among the most highly upregulated. While IL-4 caused an upregulation of these transcripts inside exosomes, artificial overexpression of miRNA target sequences caused an accumulation in P-bodies and a reduction from exosomes [48]. Again, this could provide further evidence of exosomes serving as a disposal mechanism for overly abundant miRNA levels.

### 4.3. Neuroinflammation

Various miRNAs have been implicated in neuroinflammation and neurological diseases such as Parkinson’s disease, Alzheimer’s disease, amyotrophic lateral sclerosis, and depression [49]. Astrocytes stimulated with the pro-inflammatory IL-1β both increased exosomal secretion as well as altering the miRNA content within the exosomes [50]. While certain exosomal miRNAs were increased from baseline, a subpopulation of miRNA, including let-7d, miR-126, miR-130b, miR-139-5p, and miR-141-3p, was only present in the IL-1β stimulated group [50]. This means that this population of five miRNAs was exclusively shuttled into exosomes due to the pro-inflammatory conditions. Through target scan predictions, the authors identified several potential locations where these upregulated miRNAs could target mRNAs involved in apoptosis and cell death [50].

While these miRNAs are implicated in the progression towards cell death, other groups of miRNAs have demonstrated the ability to protect against neuroinflammation. Subarachnoid hemorrhage (SAH) cause neuroinflammation leading to early brain injury [51]. As a proposed treatment, exosomes experimentally loaded with miR-193-3p and delivered to a mouse model of SAH caused a reduction in HDAC3 and a subsequent increase in acetylated-p65 levels [52]. Acetylation causes p65 to remain in the inactive form and prevents it from activating NF-κB [53]. Thus, miR-193-3p has the ability to prevent NF-κB activation subsequently reduce NF-κB-mediated inflammation. Additionally, miR-193-3p delivery also caused a reduction of pro-inflammatory mediators Caspase-3, IL-1β, IL-6, and TNF-α, further attenuating the inflammatory response [52]. Ultimately, this miR-193-3p exosomal treatment reduced brain edema and improved neurological scores in the mouse models of SAH [52].

### 4.4. Diabetes Mellitus

DM is characterized by high levels of circulating glucose and is associated with endothelial damage and dysfunction [54,55]. Furthermore, DM has been shown to increase total levels of MV released from various cell types, raising the question of whether endothelial cells might also be involved in this MV production [56,57]. Within this population of elevated MVs, hyperglycemic conditions in vitro caused a significant reduction in MV-derived miR-126 and miR-26a [58]. These results have been strengthened by other reports confirming a reduction in MV-derived and AB-derived miR-126 under hyperglycemic conditions when compared to normal controls [59]. However, alternative reports have identified an increase in miR-126 within plasma-derived EVs from human diabetic neuropathy patients [60]. The reduction of MV-derived miR-126 and elevation of EV-derived miR-126 hint at the possible mechanistic differences between sorting into the various types of extracellular bodies.

MVs have been shown to deliver miRNAs to endothelial progenitor cells (EPCs) and induce regulation over the recipient cell [61]. Specifically, delivery of miR-126 to damaged EPCs aids in the recovery process and reverses any impairment [61]. Further, EV-mediated delivery of miR-126 improved endothelial barrier function in an in vitro model [60]. Functionally, miR-126 has been shown to protect endothelial cells against H/R mediated damage, while miR-26a protects endothelial cells against ischemia-reperfusion injury [62,63]. Finally, miR-126 inhibits expression of vascular cell adhesion molecule 1 (VCAM-1), which functions to bind leukocytes and promotes inflammation [64]. Thus, the hyperglycemic-mediated downregulation of miR-126 and miR-26a inside MVs might reflect a concomitant decrease in cytoplasmic miR-126 expression, which could be partly driving the pathogenesis. This downregulation could eliminate the protective functions as well as stimulate VCAM-1. Higher VCAM-1 would promote the endothelial inflammation associated with DM. Collectively, these results point towards a regulatory mechanism whereby hyperglycemia selectively alters the packaging of miR-126 and miR-26a into MVs and contributes to the pathogenic process.

### 4.5. Cancer

A growing hypothesis regarding cancer suppression theorizes that a select population of miRNAs are secreted from healthy cells that provide growth-suppressing signals to nearby cancerous cells [65]. Syndecan-1 is a cell surface heparan sulfate proteoglycan and is known to have functions in cancer cell signaling, such as in multiple myeloma and breast cancer [66,67,68]. Analyzing exosomes from A549 cancer cells expressing syndecan-1 showed an upregulation of 43 miRNAs and a downregulation of 91 miRNAs when compared to exosomes from syndecan-1 deleted cells [69]. Of the upregulated miRNAs, exosomal expression of has-miR-485-3p was upregulated by 184-fold. In a separate report, miR-485-3p has been shown to suppress cancer growth and was found to be downregulated in breast cancer [70]. Further, A549 cells cultured with exosomes isolated from syndecan-1 expressing cells demonstrated decreased rates of proliferation compared to exosomes from syndecan-1 deficient cells [69]. Collectively, this data suggests that syndecan-1 plays a role in selectively packaging has-miR-485-3p into exosomes. These has-miR-485-3p-enriched exosomes then exert anti-tumorigenic effects over nearby cells.

As mentioned earlier in this review, several mechanisms of miRNA packaging have instead been implicated in cancer progression including MVP, nSMase2, and Vps4A [27,36,40]. However, other mechanisms have also been implicated in selective miRNA packaging into exosomes to cause tumorigenesis. In particular, tumor-derived exosomes contain the miRNA processing enzyme Dicer, which appears to actively convert pre-miRNAs to mature miRNAs within the exosomes [71]. Dicer was not found in exosomes derived from normal cells, and thus, pre-miRNA conversion to miRNA was also absent [71]. This Dicer-dependent mechanism potentially contributes to cancer progression. Healthy MCF-10A cells from human mammary glands exposed to exosomes produced by breast cancer MDA-MB-231 cells caused increased survival and proliferation [71]. However, deletion of Dicer in tumor-derived exosomes caused a reduction in the growth of MCF-10A cells [71]. This suggests that tumors have the ability to convey pro-tumorigenic signals to nearby cells through exosomes via a potential Dicer-dependent mechanism.

Cholangiocarcinoma (CCA) is a rare form of bile duct cancer also implicated in miRNA sorting [72]. Proteomic analysis of EVs derived from CCAs revealed differential expression of 95 proteins relative to control while EVs from hepatocellular carcinomas (HCC) revealed 98 dysregulated proteins relative to control [73]. Furthermore, analysis of bile-derived EVs from CCA patients revealed an upregulation of miR-191, miR-486-3p, and miR-1274b [74]. In addition to the dysregulated EVs, other reports have demonstrated a downregulation of miR-195 within the CCA cells [75]. Treating CCA cells with miR-195-loaded EVs restored expression and reduced growth and invasiveness [75]. This suggests that CCA cells are able to selectively remove miR-195 from the cytosol, but sheds light on potential EV-based therapy.

Significant work has also been conducted on examining circulating exosomal miRNAs in ovarian cancer (OC). Initial studies into OC identified a correlation between tumor miRNAs and exosomal-derived miRNAs [76]. Specifically, miR-21, miR-141, miR-200a, miR-200c, miR-200b, miR-203, miR-205, and miR-214 were upregulated in both OC tumor cells and OC-derived exosomes. In additional studies of plasma-derived exosomes from human OC patients, miR-205-5p, miR-145-5p, miR-10a-5p, miR-346, and miR-328-3p were all found to be upregulated [77]. Interestingly, in a meta-analysis study of exosomal miRNAs from solid tumors, poor prognosis was associated with upregulated miR-21, miR-200a, miR-200b, miR-200c, and miR-203 [78]. While this meta-analysis was not specific to OC, it indicates that some of the upregulated exosome-derived miRNAs from OC patients could be used as biomarkers of disease progression.

### 4.6. Heart Disease and Atherosclerosis

In response to cardiovascular stressors such as hypertension, the heart can undergo hypertrophic remodeling [79]. While the mechanism behind cardiac remodeling involves many factors, fibroblasts are essential to cardiac hypertrophy [80]. Artificially treating cardiomyocytes with media depleted of fibroblast-derived exosomes produced no cardiomyocyte hypertrophy, suggesting that fibroblast-derived exosomes play a critical role in cell growth [81]. The authors further found that certain miRNAs could mediate this hypertrophy. Transfecting fibroblasts with pre-miR-21 caused a resultant increase in miR-21 expression in cardiomyocytes after 72h [81]. Finally, transfection of fibroblast-derived exosomes enriched in miR-21 produced a resultant hypertrophy of the cardiomyocytes [81].

Atherosclerosis is widely known to be the driving force behind coronary artery disease and high levels of oxidized LDL are a significant risk factor for atherosclerosis development and progression [82]. Recently, it has been demonstrated that oxidized LDL (oxLDL) has the ability to selectively shuttle miRNAs into MVs [83]. Specifically, miR-92a-3p, miR-222-3p, and miR-26a-5p were selectively sorted into MVs through an oxLDL-dependent mechanism. In addition to oxLDL, IL-6 is a cytokine known to drive endothelial dysfunction and atherosclerosis [84]. Treating endothelial cells with IL-6 also caused increased miR-92a-3p sequestration into MVs [83]. The authors further demonstrated that oxLDL increased STAT3 phosphorylation and potentiated miR-92a-3p shuttling into MVs [83]. In mouse models, miR-92a-3p inhibition caused a reduction of aortic atherosclerotic lesions size, suggesting that miR-92a-3p is indeed involved in the propagation of atherosclerosis [85]. Thus, the oxLDL-STAT3 mechanism serves to shuttle miR-9sa-3p into MVs and promotes atherosclerosis.

Other miRNA sorting mechanisms have been found to promote atheroprotection. In particular, miR-143/145 are downregulated in atherosclerosis and have been shown to regulate smooth muscle cell (SMC) differentiation [86]. Additionally, aorta SMC differentiation is partially controlled by a transcription factor known as Krüppel-like Factor 2 (KLF2) [87]. Thus, it is reasonable to hypothesize a connection between KLF2 and miR-143/145 expression. KLF2 overexpression has recently been shown to selectively transfer miR-143/145 into endothelial EVs and that these miR-143/145-enriched EVs can be transferred to SMCs [88]. Interestingly, KLF2 overexpression in vivo caused a reduction in fatty lesions within the aortas of mice [88]. Overall, this suggests that KLF2-induced packaging of miR-143/145 into EVs exerts regulatory control over the atherosclerotic process in SMCs and helps prevent atherosclerosis propagation. An overview of this mechanism and other diseases involving miRNA-derived EV mechanisms is listed in Table 2.

## 5. Discussion

Exosomes, MVs, and ABs are all generated via independent mechanisms, but all are known to contain miRNAs. Research into EVs has expanded rapidly in recent years, largely due to newly found regulatory mechanisms whereby cells can selectively control their miRNA cargo. In order to selectively sort miRNA, RBPs such as hnRNPA2B1, Ago2, YBX-1, MEX3C, MVP, and the La protein all appear to bind miRNAs and facilitate their transfer into EVs. For many of these RBPs, the literature has recently begun to identify specific miRNA-binding motifs capable of exerting selectivity over the miRNAs shuttled into EVs. However, the specifics of these mechanisms are still largely unknown. Alternatively, certain membrane proteins such as Cav-1 nSMase2 and Vps4A have been implicated in EV biogenesis, and in the process have been shown to selectively shuttle miRNAs. Finally, certain disease states such as chronic lung disease, immune response, neuroinflammation, diabetes mellitus, cancer, and heart disease can cause dysregulation over EV-miRNA content. In some cases, selective shuttling of miRNAs into EVs has been directly implicated in the pathological process of the disease, as is the case with miR-92a-3p and atherosclerosis. However, other research has simply demonstrated differential expression of EV-derived miRNAs without a clear mechanism of pathogenic control over the disease, as is the case with miR-320a and miR-221 over hyperoxia.

Despite the knowledge that cells can selectively sort miRNA into EVs, the purpose of this sorting is still under debate. One overarching idea theorizes that EVs are secreted from parent cells to have a regulatory influence over daughter cells. Evidence has already shown that secreted EVs can be endocytosed by daughter cells and alter the mRNA or miRNA content of those cells. However, another main theory indicates that cells might secrete EVs to eliminate unnecessary RNAs, including miRNAs. While cells still could be selectively sorting miRNAs into EVs for export, the purpose would simply be for garbage disposal rather than complex signaling. In reality, cells likely use these miRNA sorting mechanisms for both signaling and disposal, but more work needs to be conducted to determine the ultimate function. Regardless of the reason that cells employ miRNA packaging, evidence has demonstrated an ability to harness this clinically. In this review, we have shown that artificially delivering EV-mediated miRNAs is beneficial in reducing neuroinflammation after SAH. This potential treatment opens the door for future artificial EV-mediated therapy.

It is important to note that there are some potential limitations to research into miRNA sorting mechanisms. Some mechanisms have been quite heavily researched, such as is the case with hnRNPA2B1 and Ago2. However, there are still relatively few studies on other miRNA sorting mechanisms, such as the MVP or La protein mechanisms. As miRNA sorting is still a relatively novel field, it is possible that the current body of literature only represents studies that have reported positive findings, especially on the less well researched mechanisms. Additionally, miRNA sorting is a complicated phenomenon likely requiring a cascade of protein-mediated events. Thus far, research has identified certain proteins associated with upregulated or downregulated miRNA levels within EVs, but specific step-by-step mechanisms are largely unreported. Therefore, it is difficult to determine whether the sorting proteins reported in this review are part of independent sorting mechanisms, or whether they all play a role in a unified larger picture sorting mechanism.

To best propel this field forward, future research should be directed towards strengthening and verifying the current findings. As mentioned, certain sorting mechanisms have little research and need additional findings to be able to make stronger conclusions. Once these sorting mechanisms have been thoroughly established, more in vivo experiments must be conducted. Most work until this point has focused on in vitro studies, but something as complicated as EV-mediated miRNA signaling must be conducted in vivo to allow for more definitive conclusions. Finally, research can aim to manipulate the miRNA sorting mechanisms listed in this review to alter the miRNA expression of naturally secreted EVs with the ultimate goal of providing therapy.

## Figures and Tables

**Table 1:** List of miRNA sorting mechanisms regulated by RNA-binding proteins.

RNA-Binding Protein	Mechanism	Reference
Heterogeneous nuclear ribonucleoprotein A2B1	Binds miR-198 and miR-601 via potential GGAG/UGCA motifs to load into exosomes	[9]
	Binds miR-17 and miR-93 via potential AGG/UAG motifs to load into MVs	[10]
Synaptotagmin-binding cytoplasmic RNA-interaction protein	Binds miR-3470a and miR-194-2-3p via potential GGCU motif to load into exosomes	[14]
Argonaute 2	Loads let-7a, miR-100, and miR-320a into EVs through KRAS-MEK-ERK signaling pathway	[18]
Y-Box Binding Protein 1	Binds miR-133 and miR-223 to load into exosomes	[22,23]
MEX3C	MEX3C combines with AP-2 and is involved in exosome biogenesis and sorting of miR-451a	[25]
Major Vault Protein	MVP shuttles miR-193a into exosomes	[27]
La protein	La protein shuttles miR-122 into EVs	[29]

**Table 2:** List of diseases and the associated effect on EV miRNA.

Disease	miRNA Involved	Reference
Chronic Lung Disease	Hyperoxia-treated lung epithelial cells caused an upregulation of miR-320a and miR-221 in MVs	[45]
	CLD causes elevated serum EV levels of miR-21 in human infants	[46]
Immune Response	IL-4 causes upregulated miR-138-5p and miR-149-5p in BMDM-derived exosomes	[48]
Neuroinflammation	Astrocytes stimulated with IL-1β increased exosomal levels of let-7d, miR-126, miR-130b, miR-139-5p, and miR-141-3p	[50]
	Exosomal miR-193-3p delivered to a SAH mouse caused a reduction in HDAC3 reduced inflammation	[52]
Diabetes Mellitus	Hyperglycemia causes a reduction in MV and AB-derived miR-126 and MV-derived miR-26a	[58,59]
	Diabetic neuropathy patients demonstrate increased miR-126 within plasma-derived EVs	[60]
	EV-mediated delivery of miR-126 improved endothelial barrier function and aids in EPC recovery	[60,61]
Cancer	In A549 cells, Syndecan-1 expression caused a 184-fold upregulation of exosome-derived has-miR-485-3p, which has been shown to suppress cancer growth	[69,70]
	Tumor-derived exosomes Dicer, which converts pre-miRNAs to mature miRNAs to promote proliferation	[71]
	Bile-derived EVs from CCA patients have upregulated miR-191, miR-486-3p, and miR-1274b and downregulated miR-195 within the CCA cells	[74,75]
	miR-21, miR-141, miR-200a, miR-200c, miR-200b, miR-203, miR-205, and miR-214 are upregulated in both OC tumor cells and OC-derived exosomes, while miR-205-5p, miR-145-5p, miR-10a-5p, miR-346, and miR-328-3p were all found to be upregulated exclusively in OC-derived exosomes	[76,77]
Heart Disease and Atherosclerosis	Fibroblast-derived exosomes mediate cardiac hypertrophy via potential miR-21 dependent mechanism	[81]
	miR-92a-3p, miR-222-3p, and miR-26a-5p are selectively sorted into MVs through an oxLDL-dependent mechanism	[83]
	KLF2 assists with packaging miR-143/145 into EVs for atheroprotective effect of SMCs	[88]
